# Bundle of care taking into account time to improve long-term outcome after cardiac arrest

**DOI:** 10.1186/s13054-018-2128-4

**Published:** 2018-08-15

**Authors:** R. Jouffroy, B. Vivien

**Affiliations:** Intensive Care Unit, Anaesthesiology department and SAMU of Paris, Hôpital Universitaire Necker – Enfants Malades, Assistance Publique - Hôpitaux de Paris, Université Paris Descartes, 149 Rue de Sèvres, 75730 Paris Cedex 15, France

Gough and Nolan [1] recently reviewed the rationale for using adrenaline in the treatment of cardiac arrest. As underlined by the authors, adrenaline has been recommended for cardiac arrest treatment since the 1960s, despite a lack of proof in animal and humans studies [1]. Adrenaline induces vasoconstriction by stimulating alpha receptors and increases tissue pressure perfusion, especially at the cerebral and coronary levels, that is associated with return of spontaneous circulation (ROSC) [2]. However, discrepancies exist between pathophysiological objectives and clinical results concerning adrenaline’s macro- and microcirculatory effects, leading since a few years to discussions on the potential negative action of adrenaline by itself [1].

Nevertheless, we believe that even if adrenaline administration remains one of the key aspects of the treatment of cardiac arrest in order to preserve tissue pressure perfusion, to improve long-term outcomes it seems essential to consider cardiac arrest resuscitation as a “bundle of care”, as similarly described for sepsis [3]. From this point of view, time is a much more important parameter than it is for sepsis: the time scale is not hours but minutes and strongly impacts not only short- but also long-term outcomes. Further studies should therefore define the components of this bundle of care and incorporate them in the fifth link (i.e., post-resuscitation care) of the chain of survival, even in the prehospital field when appropriate [4]. Regarding these components, cerebral protection should be emphasized and precociously integrated after cardiac arrest resuscitation, probably within the first minutes after ROSC. In our opinion, three targets appear important with regard to this bundle of care. Firstly, sedation should be started as soon as possible in order to put the brain to rest. Secondly, blood pressure should be optimized, avoiding both cerebral hypoperfusion due to post-resuscitation syndrome and, conversely, hyperperfusion, which could lead to cerebral edema. Thirdly, oxygen management is essential, and hyperoxia should be fought as soon as ROSC has occurred [5].

Finally, even if administration of adrenaline is a key aspect of ROSC, it is not sufficient alone to increase short- and especially long-term survival after cardiac arrest. A true bundle of care, including adrenaline, cerebral protection, and mostly a time schedule for the different therapeutics, should be implemented complementarily to the classic chain of survival.
